# Frequent Use of Emergency Departments by the Elderly Population When Continuing Care Is Not Well Established

**DOI:** 10.1371/journal.pone.0165939

**Published:** 2016-12-14

**Authors:** Jacopo M. Legramante, Laura Morciano, Francesca Lucaroni, Francesco Gilardi, Emanuele Caredda, Alessia Pesaresi, Massimo Coscia, Stefano Orlando, Antonella Brandi, Germano Giovagnoli, Vito N. Di Lecce, Giuseppe Visconti, Leonardo Palombi

**Affiliations:** 1 Department of Medical Systems, University of Rome Tor Vergata, Foundation Policlinico Tor Vergata, Rome, Italy; 2 Department of Biomedicine and Prevention, University of Rome Tor Vergata, Rome, Italy; 3 Sanitary Director, Foundation Policlinico Tor Vergata, Rome, Italy; 4 Emergengy Department, Foundation Policlinico Tor Vergata, Rome, Italy; University of Bologna, ITALY

## Abstract

**Introduction:**

The elderly, who suffer from multiple chronic diseases, represent a substantial proportion of Emergency Department (ED) frequent users, thus contributing to ED overcrowding, although they could benefit from other health care facilities, if those were available. The aim of this study was to evaluate and characterize hospital visits of older patients (age 65 or greater) to the ED of a university teaching hospital in Rome from the 1st of January to the 31st of December 2014, in order to identify clinical and social characteristics potentially associated with “elderly frequent users”.

**Material and Methods:**

A retrospective study was performed during the calendar year 2014 (1^st^ January 2014 – 31^st^ December 2014) analyzing all ED admissions to the University Hospital of Rome Tor Vergata. Variables collected included age, triage code, arrival data, discharge diagnosis, and visit outcome. We performed a risk analysis using univariate binary logistic regression models.

**Results:**

A total number of 38,016 patients accessed the ED, generating 46,820 accesses during the study period, with an average of 1.23 accesses for patient. The elderly population represented a quarter of the total ED population and had an increased risk of frequent use (OR 1.5: CI 1.4–1.7) and hospitalization (OR 3.8: CI 3.7–4). Moreover, they showed a greater diagnostic complexity, as demonstrated by the higher incidence of yellow and red priority codes compared to other ED populations (OR 3.1: CI 2.9–3.2).

**Discussion:**

Older patients presented clinical and social characteristics related to the definition of “elderly frail frequent users”. The fact that a larger number of hospitalizations occurred in such patients is indirect evidence of frailty in this specific population, suggesting that hospital admissions may be an inappropriate response to frailty, especially when continued care is not established.

**Conclusion:**

Enhancement of continuity of care, establishment of a tracking system for those who are at greater risk of visiting the ED and evaluating fragile individuals should be the highest priority in addressing ED frequent usage by the elderly.

## Introduction

The chronic overcrowding of Emergency Departments (ED) in the last decades, along with insufficient availability of inpatient beds remains one of the main Public Health and National Health Systems critical points. Prolonged wait times and poor outcomes for patients are strictly associated with this crisis. Other important consequences of overcrowding include a general perception of being rushed by emergency physicians and staff, increase in the number of ambulance diversions, and a higher frequency of patients leaving the ED without being seen (LWBS) [1–3]. The impact of overcrowding on public health and quality of care in the ED has received national attention, prompting urgent calls for reforms. Although there is not a unique cause for this phenomenon, it seems that the primary role is actually played by a lack of access to primary care services and, overall, by an inadequate development of continuing care, especially for a group of patients described as “frail”.

Based on these findings, we decided to explore the concept of frailty regarding the use of ED services. Frailty is a status of extreme vulnerability to endogenous and exogenous stressors exposing the individual to a higher risk of negative health-related outcomes, and may represent a transition phase between successful aging and disability. Fried et al [4]) exhaustively defined frailty as a biological syndrome featured by cumulative declines in different physiological systems and resulting in a loss of reserves and resistance to external stressors.

Among patients accessing the Emergency Department every year, there are individuals with repeated visits, usually 4 or more per year, the so-called “frequent users” [5–9]. This group of patients, besides greatly contributing to ED overcrowding, are usually portrayed as a costly population that often abuse or misuse ED services [10]. However, no definitive data is presently available. Far from being a homogenous group, frequent ED users likely represent a number of different distinct groups, who have not been thoroughly defined, even though anecdotal reports seem to suggest that repetitive accesses are directly correlated with age. A possible hypothesis is that older patients might represent a substantial proportion of ED frequent users because of the increasing number of chronic diseases contributing to increased risk of functional decline thus leading to frailty, although to date scientific literature is inconclusive. Therefore, older patients require significantly more emergency care resources than younger adults. This means both higher number of visits and the use of more complex resources, due to the higher number of existing co-morbidities [11–14].

There is widespread agreement that structural changes are needed to improve health outcomes for older people and to simultaneously reduce National Health System costs [15]. The perception that there is an increasing number of ED geriatric frequent users and the lack of scientific literature on this topic prompted us to evaluate the characteristics of this emerging phenomenon: the elderly as ED “frequent users”. Consequently, the aim of this study was to evaluate and characterize visits of older patients (age 65 or greater) to the Emergency Room of a University teaching hospital in Rome from the 1st January to the 31st December 2014, in order to identify clinical and social characteristics potentially related to the definition of “elderly frequent users”.

## Methods

### Population, criteria and variables

We analyzed all ED admissions to the University Hospital of Rome Tor Vergata during the calendar year of 2014, focusing on frequent users and patients aged ≥ 65 years. The hospital is a first level Department of Emergency (DEA), reference point (Hub) for specific diseases (pathologic conditions such as trauma and neuro-trauma, cardiologic emergencies and stroke), with neither obstetric nor pediatric units.

According to the Italian Privacy Legislation (n. 196/2003), and Resolution n. 85/2012 of the Guarantor for the protection of personal data, data concerning health status may be used in aggregate form and may be processed for epidemiological research purpose in scientific studies and the Institutional Review Board approved this study. No identifiable personal data were used for this study. Data extraction was performed from GIPSE information systems and the local database, using a subgroup of ICD-9 CM codes, specifically designed for the ED following Guidelines for the Lazio Region [16].

Frequent users (FU) were defined as patients with four or more visits to the ED. Variables collected included age, triage code, arrival data, discharge diagnosis, and visit outcome. Triage codes were assigned according to 4 triage categories, assessed by skilled-nursing staff based on Italian guidelines [17]: white tag identified a non-urgent condition; green tag, a less urgent condition/low priority; yellow tag, urgent, potentially life-threatening emergency conditions; and a red tag identifying a very critical, immediately life-threatening emergency condition. The closest matching categories from ICD-9-CM and ICD-10-CA codes were used to group ED visit diagnoses into major clinical categories and diagnostic clusters.

Inclusion criteria included all accesses to ED during the calendar year 2014. Patients with medication codes, who accessed the ED for drug prescriptions were excluded. The estimated cost for each patient who attended the emergency room was calculated by dividing the total amount refunded by the Lazio Region to the ED of the Policlinico Tor Vergata by the number of accesses billed by the hospital for Emergency Department visits.

### Study design

#### This was a single-center retrospective study

Data analysis was performed with IBM SPSS (Illinois, Chicago, v. 23). Means, medians, SD and interquartile ranges were calculated for quantitative variables. T-Test on the means (and associated Levin tests on variance) was used to evaluate differences between subpopulations. Descriptive analysis included frequencies and percentages for qualitative variables; median, means and standard deviations for quantitative variables. Risk analysis using univariate binary logistic regression models was performed.

## Results

A total number of 46,820 patient visits occurred during the study period. The ED was accessed by a total of 38,016 users, with a mean age of 49.6 ± 21.6 years of age. The main characteristics of the overall population are reported in Table 1.

**Table 1:** Characteristics of the overall population.

Overall population	38,016 patients	
Aged population	10,388 patients	
Mean age (SD)	49,6 ± 21,6	
Median (IQ)	48 (32–67)	
*N* of ED visits in 2014	1	31,852
	2	4,590
	3	1,044
	≥ 4	530
*N* of hospital admissions in 2014	7,023	

The most frequent triage code in the overall population was green (59%), followed by yellow (26%), white (10%) and red (3%). The main diagnoses were chest pain, kidney colic, low back pain, syncope/ loss of consciousness, and abdominal pain. Home discharge occurred in 56% of cases, hospitalization in 17% and Left Without Being Seen (LWBS) in 15% as shown in S1 Fig.

Frequent users (4 or more access per year) constituted 1.4% of 38,016 of the ED population, although they accounted for nearly 6% (2406 accesses) of the total number of ED accesses (46,820). The mean age was higher in frequent users than in the non-frequent users as shown in Table 2.

**Table 2:** Mean age and T-test of Frequent users vs non-frequent users.

Frequency of accesses	Total number of accesses	Mean AGE	Standard Deviation
≥ 4	2409	56	19.4
< 4	44355	49	21.8
*t-test FU VS NON-FU*	10,01 (*p*<0.001)	

Mean age of Frequent Users (FU; 4 or more accesses per year) vs non-Frequent Users (< 4 accesses per year). T-test results explained the difference in the mean age between the group of FU and non-FU.

However, triage codes and main diagnoses were similar in both groups. Outcomes were also different: hospitalization occurred in 22% of frequent users, five-percentage points higher than that of the overall population. LWBS happened in 18% of cases (15% in the attending population). Considering the economic reports of our hospital, the average cost of each access to the emergency department accounted for about € 282, therefore the total cost for frequent users was approximately € 627,000 per year. Considering the high incidence of admissions for this group, with an average stay of five days, the additional cost amounted to almost € 350,000.

Elderly patients were responsible for 25% of patient visits during the calendar year of 2014: 10,388 patients for 13,488 visits. Although code green was the most frequently detected triage code (46.5% of cases), triage codes red and yellow were significantly higher in the elderly than in the overall population (8.5% and 39% respectively in the elderly versus 3% and 26% in the general population). Diagnoses also differed: loss of consciousness and syncope, chest pain, stroke, pneumonia and atrial fibrillation were the five most common conditions of older people accessing the ED (Table 3).

**Table 3:** Discharge diagnosis among population visiting the ED.

DISCHARGE DIAGNOSIS		
Overall Population	Frequent Users	Older Adults
Other Chest Pain	Other Chest Pain	Syncope And Loss Of Consciousness
Kidney Colic	Some Kind Of Malaise	Other Chest Pain
Low Back Pain	Epigastric Abdominal Pain	Unspecified Transient Cerebral Ischemia
Unspecified Chest Pain	Unspecified Chest Pain	Pneumonia
Syncope And Loss Of Consciousness	Low Back Pain	Unspecified Chest Pain

Among those accessing the ED, patients aged 65 or greater were hospitalized in almost 50% of cases while this percentage decreased to 19.7% in younger individuals. Overall, increased age accounted for the majority of admissions. Among frequent users, older individuals were most frequently hospitalized (data not shown). Older patients had an increased risk of becoming frequent users (OR 1.5; CI (*Confidence Interval*) 1.4–1.7), to receive a red or yellow triage code (OR 3.1; CI 2.9–3.2) and to be hospitalized (OR 3.8; CI 3.7–4), when compared to the younger population. Consistently with previous data, *frequent users* have an increased risk of hospitalization of 40% (OR 1.4; CI 1.2–1.5) and of Left Without Been Seen (OR 1.36; CI 1.22–1.52) compared with non-frequent users.

Considering as outcomes only hospitalization and home discharge, the association between age and risk of hospitalization significantly increased (OR 5.2; CI 4.9–5.5) as provided in S2 Fig.

As highlighted in S1 Fig, the percentage of deaths in the ED was 0.5% in the overall population, 0.2% among frequent users and 1.5% in older adults.

## Discussion

As outlined in the results section, older individuals and frequent users had a significantly higher number of ED accesses, contributing to the overcrowding of emergency departments [18–20]. Crowding is a major issue for Italian EDs, even if a decreasing trend over the last 5 years has been observed. The last report from SIMEU (Italian society of Emergency Medicine), attributes overcrowding of the ED to elderly patients attending hospitalization, according to the “output” mechanism of overcrowding reported by Asplin at al [21]. Some interventions have been introduced in the attempt to limit ED overcrowding, such as ticket for white tag connected to the institution of a dedicated box, and the introduction of “fast-track system” for patients with a green code who needed specialist visits (ophthalmology, otolaryngology and dermatologists for example). These interventions should reduce the number of non-urgent visits and length of stay for these patients in the ED.

Nevertheless, overcrowding still remains one of the most important problems of our ED. Therefore, we need to deal with the last mechanism of ED overcrowding, the “output”, which addresses the capability of a hospital to make available beds in order to discharge patients thus decongesting the emergency room. It is also true that the flows from the ED to the Hospital Departments, are likely to prolong the waiting lists for elective hospitalization [22–25].

The output mechanism comprises two important issues: the availability of beds in the hospital, and the availability of continuity of care for patients after hospital discharge who can’t return home. Both circumstances were rare within our hospital patients. In fact, despite the estimated national demand and regional hospital and community care, the assistance offered in the Local Health Unit Rmb (now Rm2) remains far below minimum limits, as noted in Table 4.

**Table 4:** Difference between demand and offer on primary care.

	Demand	Offer	Ongoing Offer	Difference
Intensive Residence	162	60	20	- 82
Extensive Residence	727	-	20	- 707
Manteining Residenzial type A	5982	2349	-	- 3633
Nursing Home	2692	471	-	- 2221
Psychiatric Beds	32	16	16	- 16
Home Care	5155	2700		- 2455

The characteristics of the territory in which our hospital is situated is important to consider, in order for adequate services for special patients to be planned. The Local Health Unit RmB (now HLU Rm2) is the biggest in the Lazio Region, with about 745,000 inhabitants. The percentage of aging people is 19% and there is a strong component of immigrants (15.5%, twice the national data). Moreover, the socioeconomic status (ranging from 0 –no social/economic problems to 100—maximum economic problems) of this population is the lowest in the city, 73.6 of 100 versus a mean value of 50 for Rome. In the last census, 4000 families lived with disabilities. This was the highest proportion of adults in Rome requiring economic assistance [26].

However, the most important data is the lack of primary care facilities for post-acute care, such as nursing homes or rehabilitation clinics, and a system for continuation of care, which, along with the socio economic support, would address the needs of patients in the ED [26,27]. This general frail condition in our territory might explain the high rate of hospitalizations in our hospital for elderly patients. Indeed, the risk of hospitalization in older adults demonstrated in our study is one the highest ever reported in the literature (Table 5).

**Table 5:** Risk of hospitalization among elderly patients as described in the literature.

Authors	Year	Study design	Older adults’ hospitalization	Odds Ratio
Aminzadeh F.	2002	Systematic review	32–51%	2.5–4.6
Yim VWT.	2009	Retrospective analysis	45%	3
Lowthian JA.	2012	Retrospecive analysis	39%	3.9
Keyes DC.	2014	Case-control	51%	2
FRANCHI C.	2016	Retrospective analysis	21%	
**PTV**	**2014**	**Retrospective analysis**	**50%**	**5.2**

Although there are many studies that explain how the elderly and frequent users utilize the ED [5,8,9,11–14], relatively few have focused on the Italian population [27–28]. In the Lombardy region, Franchi C. et al [29] demonstrated that 24% of the elderly attended the ED (25% in the elderly frequent users). Therefore within national comparisons, our rate of hospitalization results were higher. A better organization of care could reduce the number of ED accesses and subsequent hospitalizations, thus reducing the use of both human and economic resources [28,30–32]. Our results show that more than a quarter of our patients can be considered elderly. This group of patients showed greater complexity as demonstrated by the higher incidence of yellow and red priority codes as compared to the attending population. More important is the evidence that the elderly have an increased risk of becoming frequent users and to be hospitalized when compared to their younger counterparts.

The percentage of frequent users in our ED was lower than that reported elsewhere. This might be due to the different National Health System, public versus insurance dependence, counteracting the statement that universal insurance favors high ED use [8]. Data reported in international studies, amounted the percentage of frequent users around 6–8%, however these patients constitute 21% to 28% of all ED accesses [5,8,9]. In the study of Fuda and Immekus [33] the percentage of frequent users of ED services was similar to our results (3% of frequent users), but the associated accesses were 17.6%, definitively higher than in our case series (6%). Nevertheless, frequent users in our study had a mean age of about 60, and a higher hospitalization rate when compared to the non-frequent users. Recent data confirm these results also within a different context [34]. Therefore, an effort should be made towards the introduction of clinical pathways which are able to identify these patients in the ED and correctly treat them with targeted home care protocols.

The percentage of frequent users who left our hospital without being seen was higher than that reported in the national data, supporting the hypothesis of extremely long wait times. It is unknown if these patients returned at other times or utilized other health facilities. However, even among patients < 65 years, the number of people LWBS was greater when compared to the national average, denoting an overcrowded hospital, with subsequent impact on the quality of care.

As geriatric utilization of the ED increases, due to the well-known increase in the elderly population, it is possible that the number of frequent geriatric users will grow in parallel [11,13,14]. People 65 years of age or older constitute about 20% of the Italian population. Projections suggest that by 2050 this proportion will increase to 33.2% [35]. As evidenced by available data, often an ED visit by an elderly patient is a sentinel event indicating a declining health status. Moreover, it is well recognized that the elderly who visit the ED have more frequent social and health care needs. Although two out of every three elderly individuals are unable to perform at least one of the Activities of Daily Living, functional decline and psychosocial problems are rarely recognized during ED visits [36]. In addition, geriatric patients may be admitted to the hospital not for apparent medical problems, but due to social issues. Nevertheless, many elderly individuals have an underlying acute medical problem, with mortality rates being as high as 34% [23]. Although integration of social and health care is a goal of the recent Italian Pact for Health 2014–2016, differences in social-health local networks are found in Italy [37].

It is appropriate to screen older adults for cognitive impairment, functional problems, and existing home care when they arrive in the ED in order to plan for age-specific suitable settings of care [38,39].

As reported, the ED is an important point of access for patient care. Due to the long waiting lists for regular care, the ED is often the fastest way of assessing specialist services, laboratory tests and radiological investigations. Traditional emergency medicine systems were designed to treat single acute conditions. This approach does not adequately address the complex care needs of older patients, who more often suffer from multiple and interrelated conditions. The triage system, developed for rapid prioritization and management of life-threatening conditions, has failed to identify these complexities, thus precipitating the admission of older patients [26]. Current evidence suggests that a large proportion of older individuals present risk factors for major health-related events and unmet clinical needs when they turn to the ED for assistance.

Although not conclusive, our data demonstrate that older individuals, due to their vulnerable condition, are more often seen in the ED. Although multiple tools are available for identification of elderly individuals who are at risk for adverse outcomes, these tools are often underutilized. More importantly, these tools do not completely evaluate the general conditions of these patients. For this reason, the authors suggest that a multidimensional evaluation of older patients in the ED is needed, in order to plan for appropriate settings of care and for evaluation of frailty. Frail elders, have the highest probability of suffering an adverse event. The identification of this profile is very important for decision making regarding the final patient location and discharge follow-up plans.

### Limits

Our study has some limitations. First, this is a retrospective study, for this reason we are not able to trace clinical or pharmacological characteristics of patients, beyond that reported in the computing database. A prospective study will need to evaluate these issues in the elderly that attend the ED in the future. Secondly, we only evaluated a single hospital, while in the same HLUs there are at least two other hospitals. For this reason, we are not able to determine if patients were referred to multiple sites for care. As suggested in many studies, patients who use the ED with high frequency may seek multiple sources of care, because of dissatisfaction with the treatment received. In our study the percentage of frequent users was too low to extend the results to other contexts. Nevertheless, the distribution found in previous studies indicates an incremented risk of becoming FU for people who are over 65 years of age, according to our hypothesis. On the other hand, we performed a retrospective study with the data collected from an administrative database. Therefore, we could not gather information on co-morbidities, functional status, polypharmacy and frailty.

As suggested in prior studies, a different approach to elderly patients in the ED is needed, in order to appropriately address this vulnerable population. A multidimensional evaluation, associated with hospital-based management, could fill the gap in resources for our area.

## Conclusion

According to our study, and despite the lack of available data in the literature on this topic, it appears that the major concern in the years to come will be the management of older individuals. Our hypothesis is that poor services and lack of continuity of care could explain the abnormal patterns of ED use in elderly patients. After discharge, these patients often return to the ED, highlighting the need to develop suitable ways for management of issues in this age group of patients.

Based on this data, it seems necessary to enhance continuity of care, as well as communication within the territory and the definition of a tracking system for those who are at greater risk of visiting the ED. Evaluations should identify fragile individuals as the highest priority. Coherently with the persistent lack of services in the territory, it would seem appropriate to initiate a discharge service directly managed by the hospital in order to reduce patient visits to the ED, further reducing the number of hospitalizations and the average length of stay. Finally, prospective studies on this complex issue need to better delineate the role of frailty in the elderly population.

## Supporting Information

S1 Fig Distribution of discharge diagnosis among patients attending in ED. (TIF)

S2 Fig Risk of hospitalization among the elderly considering as outcome only home-discharge and admission to the hospital. (TIF)

S1 File Minimum Data set. (XLS)
