# Supplementary figures and images for: Frequent Use of Emergency Departments by the Elderly Population When Continuing Care Is Not Well Established
Source: PLoS One. 2016 Dec 14;11(12):e0165939. doi: 10.1371/journal.pone.0165939 (PMC5156362; doi:10.1371/journal.pone.0165939)

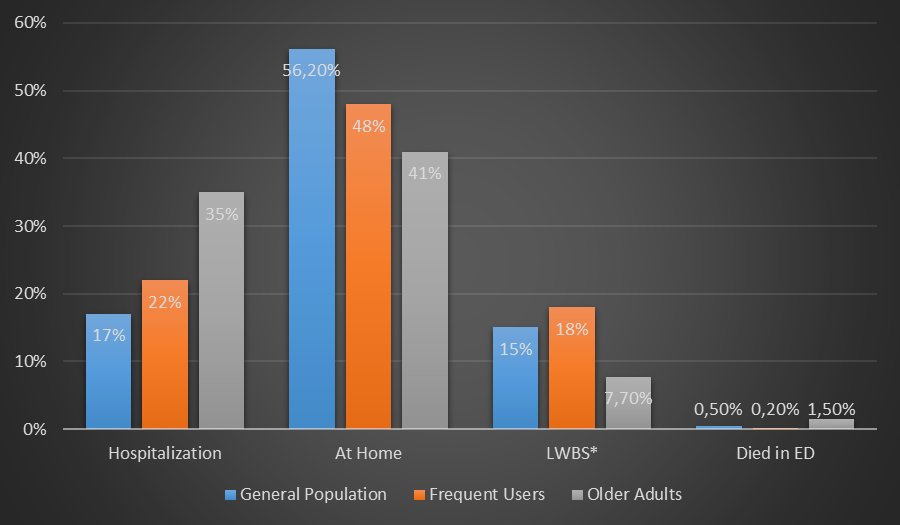

Supplement: S1 Fig — (TIF) [file pone.0165939.s001.tif]
